# List of reviewers

**DOI:** 10.2471/BLT.24.970124

**Published:** 2024-01-01

The Editorial Team would like to thank all those who gave generously of their time and expertise in reviewing the papers for the *Bulletin of the World Health Organization *in 2023. We apologize to anyone whose name has been inadvertently omitted from the following list:

## A

Abbas, Faisal

Abdelhai, Rehab

Abdulameer, Atheer

Aboushady, Ahmed

Adair, Tim

Adam, Taghreed

Adam, Mary

Addo-Atuah, Joyce

Aduo-Adjei, Kofi

Afzal, Muhammad

Aggarwal, Bhagwan

Ahmad, Abdul

Ahmad, Irshad

Alazab, Raed

Allen, Koya

Allukian, Myron

Al-Naggar, Redhwan

Aloosh, Mehdi

Amer, Faten

Amjad, Sohail

Amul, Gianna Gayle

Andersen, Karl

Anish, TS

Ansu-Yeboah, Evans

Ardal, Christine

Atuheire, Emily

Avalos Jonhston, Cristian

Aziz, Roomi

Aziz, Fatima

## B

Baek, Yeji

Bahamondes, Luis

Bahena, Alejandro

Barnes, Karen

Basaleem, Huda

Basu, Partha

Battersby, Jane

Battle, Clare

Becker, Susan

Begum, Shahnur

Beksinska, Mags

Bellingham-Young, Denise

Belsare, Aniruddha

Bettcher, Douglas

Bhandari, Dinesh

Bharti, Omesh

Bhavsar, Vishal

Bhutta, Zulfiqar

Boisson, Alix

Bollars, Caroline

Bollyky, Thomas

Bolumar, Francisco

Boniol, Mathieu

Brikci, Nouria

Brown, Ronald

Bump, Jesse

Burkholder, Taylor

## C

Caballes, Alvin

Calderwood, Claire

Callinan, Sarah

Capewell, Simon

Carpenter, Matthew

Chadha, Vineet

Chakraborty, Rhyddhi

Chang López, Roberto

Chansa, Collins

Chatzidiakou, Lia

Chen, Bin

Cheung, Yee Tak Derek

Chong, Wei

Chuki, Pem

Cieza, Alarcos

Citron, Isabelle

Coetzee, David

Cohen, Joanna

Connor, Stephen

Cornetta, Kenneth

Cowling, Benjamin

Crigler, Lauren

Curioso, Walter

Cylus, Jonathan

## D

Darvishpour, Azar

Das, Saibal

Datta, Manjula

de Witte, Luc

Deboutte, Danielle

Decouttere, Catherine

Delpeyroux, Francis

Deng, Wenlin

Der, Carolina

Devleesschauwer, Brecht

Dhawan, Dhriti

Dhillon, Ibadat

Dickmann, Petra

Dorj, Gereltuya

Drury, John

Du, Yi-Qi

du Cros, Philipp

Durbin, Anna

## E

Ebi, Kristie

Ebrahim, Shahul

Eckhardt, Christopher

Edirippulige, Sisira

Egger, Joseph

El-Kassas, Mohamed

El-Osta, Austen

Enria, Delia

Eto, Takashi

Eze, Paul

## F

Fagan, Johannes

Fakhri, Rasheed

Farmer, Jillann

Fasina, Folorunso

Ferguson, Laura

Figueras, Albert

Fleury Sattamini, Isabela

Ford, Nathan

Forsberg, Birger

Fréel, Stéfanie

Friebel, Tara

## G

Gao, Rongbao

Garba, Amadou

García-Díaz, Rocío

Gauld, Robin

Gautham, Meenakshi

Ghosh, Kanjaksha

Giersing, Birgitte

Gigante, Valeria

Giref, Ababi

Girond, Florian

Glynn-Robinson, Anna

Godman, Brian

Goldblatt, Peter

Goodman, Kenneth

Gorina, Yelena

Grassly, Nicholas

Greenbaum, Dan

Gregory, Richard

Guevara, Maria

Guidos, Brittany

Guzman-Vilca, Wilmer Cristobal

## H

HabibiSaravi, Reza

Hafner, Marco

Hands, Christopher

Hanefeld, Johanna

Hanson, Mark

Haranath, Sai Praveen

Harewood, Heather

Hartsough, Kieran

Heer, Emily

Heinmüller, Rolf

Hensher, Martin

Herrick, Kirsten

Hewitt, Victoria

Hilmers, David

Hiratsuka, Yoshimune

Homer, Caroline

Hu, Yanhong

Husain, Sara

Hwahng, Sel

## I

Ide, Nicole

Ijaz, Nadine

Irfan, Furqan

Islek, Elif

## J

Jacobs, David

Jafar, Anisa

Jafri, Hussain

Jain, Amita

Jiang, Hongbo

Jindal, Rahul

Joffe, Ari

John, Denny

Joseph Joy, Jacobs

## K

Kadam, Atul

Kahwaji, Areej

Kalita, Anuska

Kazeem, Olalekan

Kazembe, Abigail

Khan, Selim

Khosravi, Ardeshir

Knauss, Samuel

Kraay, Alicia

Krech, Ruediger

Krugman, Daniel

Kucuk Bicer, Burcu

Kuhlmann, Ellen

Kumar, Jan

Kureya, Tendayi

Kwamie, Aku

## L

Labonte, Ron

Lackland, Daniel

Lahariya, Chandrakant

Langlois, Etienne

Lankoande, Martin

Larsen, Sophia

Larson, Heidi

Lassi, Zohra

Lau, Eric

Lavorgna, Luigi

Leas, Eric

Lemiere, Christophe

Lengeler, Christian

Leye, Dessalegn Temesgen

Li, Jian

Li, Henry

Liang, Song

Lim, Lee-Ling

Lim, Audrey

Lim, Sahnah

Lionello, Lorenzo

Littler, Katherine

Liu, Xiaoyun

Llorente-Nieto, Pedro

Lokmic-Tomkins, Zerina

Lopert, Ruth

Lu, Pei-Rong

Ludolph, Ramona

Lumpkin, Murray

Luthra, Rita

Lynch, Muireann

## M

Macarthy, Joseph

Maffioli, Elisa

Malqvist, Mats

Mariotti, Silvio

Marques, Ana Patricia

Martin-Ortega, Olga

Marx, Michael

May, Win

McCormack, Valerie

McCoy, David

Mcknight, Jacob

McPake, Barbara

Meier, Scott

Michaelakis, Antonios

Miller, F. DeWolfe

Mock, Nancy

Mohammad, Yousser

Mohanty, Saroj

Moja, Lorenzo

Molyneux, Elizabeth

Morand, Serge

Morin, Sebastien

Morse, Stephen

Mouslim, Morgane

Mugabo Semahore, Jules

Murphy, Kelly

Musoke, David

Mzilahowa, Themba

## N

Nanda, Samir

Naseri Boori Abadi, Tahereh

Nasiri, Saeideh

Natarajan V, Karthik

Neves, Rita

Newton, Mark

Nikoloski, Zlatko

Nilson, Eduardo

Nola, Iskra

Núnez-Vázquez, Erick

## O

Oancea, Sanda

Obermann, Konrad

Ogoina, Dimie

Ohkado, Akihiro

Okoli, George

Olaoye, Itse

Özdemir, Vural

## P

Palacio-Mejia, Lina

Pan, Jay

Panda, Prasan

Pannenborg, Ok

Panthee, Asmita

Papanicolas, Irene

Parry, Christopher

Paulin, Sarah

Pecoul, Bernard

Pepito, Veincent

Perez Gonzalez, Maria Mercedes

Perry, Henry

Perumal, Nandita

Pike, Ian

Polak, Paulina

Polkowski, Maciej

Pollackk, Todd

Pongutta, Suladda

Prakash, Shantanu

Prasertchai, Araya

Pu, Christy

Puthoopparambil, Soorej

## Q

Qomariyah, Siti

Queiroga, Ana Catarina

Queiroz, Bernardo

## R

Rade, Kirankumar

Rahman, Mashiur

Ramesh, Nithya

Ramkumar, Vidya

Rane, Anil

Raphael, Dennis

Ratevosian, Jirair

Rawat, Pragati

Razum, Oliver

Reddy, Ravi

Remuzzi, Giuseppe

Riccò, Matteo

Richards, Heather

Ridde, Valery

Riddell, Travis

Riegel, Barbara

Rifat, Shaikh

Robert, Alexis

Rodewald, Lance

Rodriguez-Hart, Christine

Rogawski-Mcquade, Elizabeth

Rogers, Lisa

Roy, Manas

Roy, Shongkour

Roy, Nobhojit

Russo, Giuliano

## S

Sadana, Ritu

Saleem Sheikh, Mohd

Saniel, Ofelia

Sarbadhikari, Suptendra

Scafato, Emanuele

Schwalbe, Nina

Seeher, Katrin

Seng, Jun Jie Benjamin

Setel, Philip

Shaivitz, Marla

Shanquan, Chen

Shimazu, Taichi

Siddiqui, Faraz

Siddiqui, Muhammad

Silenou, Bernard Chawo

Silva, Angélica

Singh, Deependra

Sinha, Abhinav

Sinno-Tellier, Sandra

Sitas, Freddy

Siyam, Amani

Sleet, David

Sloan, Derek

Squires, Neil

Srivastava, Ambey

Ssentongo, Paddy

Steffen, Robert

Steinert, Janina

Sullivan, Richard

Sunderland, Vivian

Suthar, Amitabh

Szeto, Stephanie

## T

Talbert-Slagle, Kristina

Talwar, Shivangi

Tangcharoensathien, Viroj

Tannor, Abena

Tao, Julie

Tawar, Shabeena

Templeton, Anne

Tenner, Andrea

Tepper, Naomi

Tesfaye, Wudalew Meselu

Thomas, Marshall

Thompson, Peyton

Thong, Meow Keong

Tickell, Kirkby

Tikkanen, Roosa

Tiwana, Maida

Tjong Kim Sang, Erik

Togbo, Iza Donna

Tortosa, Fernando

Toth, Ildiko

Tripathi, Vandana

Tsai, Feng-jen

## U

Ursino, Moreno

## V

van den Ham, Rianne

VanderEnde, Kristin

Vanderlee, Lana Mae

Vapattanawong, Patama

Ventura, Deisy

Vicario-Merino, Angel

Vilcahuaman, Luis

Vincent, Jerry

von Gaudecker, Jane

Votruva, Nicole

## W

Wagley, Ujjal

Wang, Jianghao

Waning, Brenda

Wariri, Oghenebrume

Watkins, Jonathan

Wernli, Didier

Willcox, Merlin

Wolbring, Gregory

Wouters, Olivier

Wright, Stephen

## Y

Yang, Lianping

Yew, Ying Ying

## Z

Zaidi, Syed Hasan

Zeng, Wu

Zhangqi, S

Zwijnenburg, Jorrit

